# Host‐plant availability drives the spatiotemporal dynamics of interacting metapopulations across a fragmented landscape

**DOI:** 10.1002/ecy.3186

**Published:** 2020-10-07

**Authors:** Øystein H. Opedal, Otso Ovaskainen, Marjo Saastamoinen, Anna‐Liisa Laine, Saskya van Nouhuys

**Affiliations:** ^1^ Organismal and Evolutionary Biology Research Programme University of Helsinki Helsinki Finland; ^2^ Department of Biology Lund University Lund SE‐223 62 Sweden; ^3^ Centre for Biodiversity Dynamics Department of Biology Norwegian University of Science and Technology Trondheim N‐7491 Norway; ^4^ Helsinki Institute of Life Science University of Helsinki Helsinki Finland; ^5^ Department of Evolutionary Biology and Environmental Studies University of Zurich Zurich CH‐8057 Switzerland; ^6^ Department of Ecology and Evolutionary Biology Cornell University Ithaca New York 14853 USA

**Keywords:** metacommunity dynamics, multitrophic interactions, null model, plant–animal interactions, spatiotemporal dynamics, tripartite interactions

## Abstract

The dynamics of ecological communities depend partly on species interactions within and among trophic levels. Experimental work has demonstrated the impact of species interactions on the species involved, but it remains unclear whether these effects can also be detected in long‐term time series across heterogeneous landscapes. We analyzed a 19‐yr time series of patch occupancy by the Glanville fritillary butterfly *Melitaea cinxia*, its specialist parasitoid wasp *Cotesia melitaearum*, and the specialist fungal pathogen *Podosphaera plantaginis* infecting *Plantago lanceolata,* a host plant of the Glanville fritillary. These species share a network of more than 4,000 habitat patches in the Åland islands, providing a metacommunity data set of unique spatial and temporal resolution. To assess the influence of interactions among the butterfly, parasitoid, and mildew on metacommunity dynamics, we modeled local colonization and extinction rates of each species while including or excluding the presence of potentially interacting species in the previous year as predictors. The metapopulation dynamics of all focal species varied both along a gradient in host plant abundance, and spatially as indicated by strong effects of local connectivity. Colonization and to a lesser extent extinction rates depended also on the presence of interacting species within patches. However, the directions of most effects differed from expectations based on previous experimental and modeling work, and the inferred influence of species interactions on observed metacommunity dynamics was limited. These results suggest that although local interactions among the butterfly, parasitoid, and mildew occur, their roles in metacommunity spatiotemporal dynamics are relatively weak. Instead, all species respond to variation in plant abundance, which may in turn fluctuate in response to variation in climate, land use, or other environmental factors.

## Introduction

Species interactions within and among trophic levels are central to the assembly, structure, and dynamics of communities (Paine 1966, Holt 1997, Guzman et al. 2019) and, more generally, to the origin and maintenance of biodiversity (Ehrlich and Raven 1964, Stebbins 1970, Estes et al. 2011, Janz 2011). When environmental change perturbs the population dynamics of one species, the dynamics of interacting species may be directly or indirectly affected, causing a community to change. To understand the structure and dynamics of communities, we therefore need to understand the interdependence of population dynamics among interacting species across trophic levels. Plants and their associated insects and pathogens represent a large percentage of the total species in terrestrial ecosystems, and interactions among them are ubiquitous, thus providing ideal study systems for understanding the joint dynamics of interacting species (Thompson 2005).

Correlated population dynamics among plant‐associated organisms may arise in several ways. First, multiple species may respond to variation in the availability of host plants, thus creating shared dynamics without strong interactions among them. This can occur when the availability and quality of the host plant is determined primarily by abiotic environmental variation rather than by any of the plant‐associated organisms (Strong et al. 1984). If the dynamics of the host plant is determined by abiotic drivers such as large‐scale climatic fluctuations, this can lead to variation that can drive entire metacommunities (Post and Pedersen 2008, Hansen et al. 2013). Alternatively, interacting species may directly or indirectly affect each other’s dynamics.

Some interspecific effects on population dynamics are strong and thus easily detected, such as those occurring in simple predator–prey systems in the intertidal (Paine 2002), in ponds (Cottenie and de Meester 2004), and in the high arctic (Gilg et al. 2003). In many cases, however, interactions are more subtle. Both herbivorous insects and plant pathogens can affect the abundance of their host plant (Penczykowski et al. 2015) and its quality as a resource, thus setting the stage for plant‐mediated interactions (Inbar and Gerling 2008, Shikano et al. 2017). However, although interactions between herbivorous insects and/or pathogens that share host plants have been frequently detected (Strauss 1991, Van der Putten et al. 2001, Biere et al. 2002, Stout et al. 2006), and their community‐level consequences are strong (Price 1992, Bagchi et al. 2014), few studies have teased apart the influences of such interactions on the population dynamics of multiple interacting species across landscapes over time. Similarly, tritrophic interactions among plants, insects, and parasitoids have been well studied mechanistically (van Veen 2015, Kaser and Ode 2016), and their importance is evident from the perspective of trophic cascades and food webs (Hairston et al. 1960, Ripple et al. 2016, Thierry et al. 2019). The consequences of these interactions for population dynamics have been documented in controlled experiments (Maron and Harrison 1997, Cronin and Haynes 2004), in agricultural and other managed ecosystems (Murdoch 1994, Duan et al. 2015), and at single sites (Roininen et al. 1996, Price and Hunter 2015). However, beyond outbreak‐style population cycles (Turchin et al. 2003, Liebhold et al. 2012), little is known about patterns of interrelated host–parasitoid population dynamics in space across natural landscapes (Crawley 1989, van Nouhuys and Hanski 2005, Cronin and Reeve 2014), and in time as the environment changes (Boggs 2016, Kahilainen et al. 2018).

Time‐series data on potentially interacting species collected from multiple sites can be used to assess how both spatial and temporal dynamics of species depend on the presence of potentially interacting species (Yackulic et al. 2014, Rota et al. 2016, Davis et al. 2017, Ovaskainen et al. 2017*a*, Dubart et al. 2019, Fidino et al. 2019). For example, in their long‐term study of a native and an invasive snail occupying a network of several hundred ponds in the West Indies, Dubart et al. (2019) detected strong effects of patch occupancy by the potential competitor on colonization rates, suggesting reciprocal competitive effects. Analyses of time‐series data have also provided insights into interactions between native and invasive bird species (Yackulic et al. 2014), fish and amphibians in wetlands (Davis et al. 2017), and mammals in urban environments (Fidino et al. 2019). Although these studies have yielded some examples of apparent species‐interaction effects on community dynamics, very large data sets are needed to detect small effects. Progress in this area is thus hampered by the scarcity of high‐quality time‐series data for multiple species occupying a set of shared habitat patches across a landscape.

**Fig. 1:**
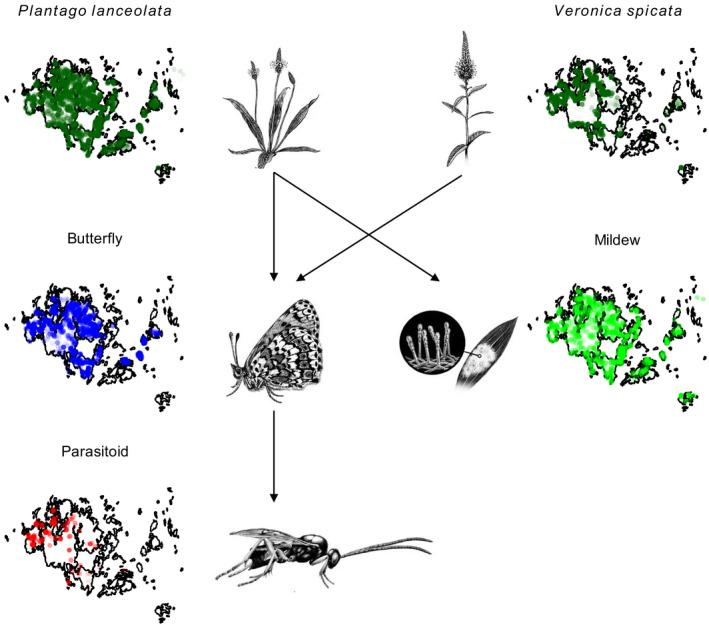
Spatial distribution of the Glanville fritillary butterfly *Melitaea cinxia*, its host plants *Plantago lanceolata* and *Veronica spicata*, its parasitoid wasp *Cotesia melitaearum*, and the powdery mildew *Podosphaera plantaginis* infecting *P. lanceolata* in the Åland islands, southwestern Finland. Color intensity indicates the proportion of years occupied for the butterfly, parasitoid, and mildew, and the relative abundance for the host plants. Arrows indicate known species interactions, including the tritrophic interaction between the host plants, butterfly, and parasitoid, and the tripartite interaction between the butterfly, mildew, and their shared host plant. [Color figure can be viewed at wileyonlinelibrary.com]

The Glanville fritillary butterfly, *Melitaea cinxia* (Nymphalidae), its host plants *Plantago lanceolata* and *Veronica spicata* (Plantaginaceae), its specialist parasitoid wasp *Cotesia melitaearum* (Hymenoptera: Braconidae), and the specialist fungal pathogen *Podosphaera plantaginis* infecting *P. lanceolata* inhabit a shared network of dry meadows and pastures in the Åland islands, in southwestern Finland (Fig. 1). They comprise a long‐term model system for metapopulation dynamics across fragmented landscapes, and all three species have been shown to persist as metapopulations with frequent local extinction and (re‐)colonization (Hanski 1999, Jousimo et al. 2014, Hanski et al. 2017). Experimental studies of individual species pairs in the system have demonstrated that these species influence each other’s fitness, and suggested that they may also affect one another’s population dynamics (Table 1).

**Table 1:** Summary of studies assessing direct and indirect interactions among the Glanville fritillary butterfly *Melitaea cinxia*, its host plants *Plantago lanceolata* and *Veronica spicata*, its parasitoid wasp *Cotesia melitaearum*, and the fungal pathogen *Podosphaera plantaginis* infecting *P. lanceolata* in the Åland islands. + and − indicate the direction of the effect of Species 2 on Species 1.

Species 1	Species 2	Mechanisms of individual interaction, effect of 2 on 1 (direction of interaction).	Population level effects
Direct interactions			
*Melitaea cinxia*	*Plantago lanceolata*	Host plant (+) [1], iridoid glycosides (+) [2, 3]	Density (+) [4, 5], Local relative abundance [6, 7]
*Melitaea cinxia*	*Veronica spicata*	Host plant (+) [1], iridoid glycosides(+) [2, 3]	Density (+) [4, 5], Local relative abundance [6, 7]
*Plantago lanceolata*	*Melitaea cinxia*	?	Density (−) [4]
*Podosphaera plantaginis*	*Plantago lanceolata*	Host plant (+)	Presence and colonization (+)
*Plantago lanceolata*	*Podosphaera plantaginis*	Plant pathogen (−)	Population growth rate (−)
*Cotesia melitaearum*	*Melitaea cinxia*	Host insect (+) [8]	Density (+) [9, 10], phenology [11]
*Melitaea cinxia*	*Cotesia melitaearum*	Parasitoid (−) [8]	Density (+) [9], phenology [11]
Indirect interactions			
*Melitaea cinxia*	*Podosphaera plantaginis*	Larval fitness (−) [12 –14]	Overwinter mortality (−)
*Cotesia melitaearum*	*Podosphaera plantaginis*	female sex bias (+ and −), brood size (+) [12, 14]	Colonization rate (+) [14]
*Cotesia melitaearum*	*Plantago lanceolata*	Iridoid glycosides (+) [15]	
	*Veronica spicata*	VOCs (+) [16]	Population size (+) [17]

Notes

References: [1] Kuussaari et al. (2004), [2] Saastamoinen et al. (2007), [3] Nieminen et al. (2003), [4] Kahilainen et al. (2018), [5] Hanski et al. (1995), [6] Hanski and Singer (2001), [7] Kuussaari et al. (2000), [8] Lei et al. (1997), [9] Lei and Hanski (1997), [10] van Nouhuys and Hanski (2002), [11] van Nouhuys and Lei (2004), [12] Karlsson Green et al. (in preparation), [13] Laine (2004*a*), [14] van Nouhuys and Laine (2008), [15] Harvey et al. (2005), [16] Pinto‐Zevallos et al. (2013), [17] van Nouhuys and Hanski (1999).

The studies summarized in Table 1 suggest that the metapopulation dynamics of each species, and thus the dynamics of the metacommunity “module” to which they belong, should depend directly and indirectly on patterns of patch occupancy by the other species. Furthermore, the dynamics of all these species may depend on the dynamics of the host plants on which they directly or indirectly depend (Fig. 1). Here, we combine for the first time the time‐series data (2000–2018) across the Åland islands, to investigate the interdependence of the dynamics of the butterfly, its parasitoid, and the mildew, and their responses to variation in the abundance of the plants *P. lanceolata* and *V. spicata*. Because there is little turnover (local colonizations and extinctions) of the plant populations (Ojanen et al. 2013), we focus our analyses on potential interactions between the insects and the pathogen, while treating the plants as an underlying driver/mediator of interactions. We approach the dynamics of the “metacommunity” mechanistically from the perspective of each focal species, as a set of potentially interacting metapopulations (Leibold et al. 2004), and do not consider higher‐level ecological properties such as species richness across trophic levels.

Our overarching hypothesis is that species interactions affect metacommunity dynamics across the landscape. If so, we expect the colonization and/or extinction probability of at least one of the focal species to differ depending on the community composition of the focal patch (i.e., the “state” of a patch indicating the local presence/absence of potentially interacting species). Because the probability of transition between any two patch states can be written in terms of the colonization and extinction rates of the individual species (Fidino et al. 2019), a detectable effect of patch state on one or more of these rates directly implies an effect on metacommunity dynamics. Focusing on the individual rates allows us to assess not only if species co‐occurrence patterns affect metacommunity dynamics, but also to pinpoint particular candidate mechanisms (e.g., the presence of species A increasing the extinction probability of species B). Metacommunities can also be described in terms of the distribution of patch states over time and across the landscape, which will depend on the sum of all species‐interaction effects on the colonization and extinction rates of individual species as well as variation in other biotic and abiotic drivers. Effects of species interactions on the colonization–extinction dynamics of individual species may or may not detectably affect the distribution of patch states, depending on the relative strength of these factors. A complementary but less mechanistic test of whether species interactions affect metacommunity dynamics is therefore to ask whether the observed distribution of patch states differs from predictions derived from models assuming “independent” dynamics of each focal species. We combine these two approaches to test whether species interactions leave a detectable signature in metacommunity dynamics.

## Materials and Methods

### Natural history of the Åland dry‐meadow metacommunity

In Åland, *P. lanceolata* and *V. spicata* grow in dry meadows, pastures, and rocky coastal areas, which occur mostly as discrete habitat patches bordered by agriculture, forest, water, roads, and human development. Scattered plants occur along roadsides. *Plantago lanceolata* occurs throughout the Åland islands, and *V. spicata* is abundant only in habitat patches in the western part of the study area (Fig. 1; Hanski and Singer 2001).

The butterfly *M. cinxia* occurs across Eurasia but is rare or extinct from most of western Europe. The larvae feed on a few species of the Plantaginaceae, and in the Åland islands feed only on *P. lanceolata* and *V. spicata* (Wahlberg 2001). *Melitaea cinxia* is univoltine in northern Europe. Adults lay eggs in clusters of about 100 in the early summer (Saastamoinen 2007). Larvae hatch and live in gregarious, mainly family, groups through the summer (Fountain et al. 2018). They spend the winter in gregarious silken nests, resume feeding in early spring, and pupate in the leaf litter in the late spring (Kuussaari et al. 2004). Several hundred habitat patches in Åland, ~15–20% of the suitable habitat patches, are typically occupied by the butterfly, with high turnover of habitat occupancy and abundance among years (Tack et al. 2015, Kahilainen et al. 2018).

In Åland, *M. cinxia* is primarily parasitized by two specialist larval parasitoids, *Hyposoter horticola* (Hymenoptera: Ichneumonidae) and *C. melitaearum*, and a generalist pupal parasitoid, *Pteromalus apum* (Hymenoptera: Pteromalidae; van Nouhuys and Hanski 2005). *Hyposoter horticola* is present throughout the landscape, and consistently parasitizes about a third of the *M. cinxia* larvae in each habitat patch (Montovan et al. 2015). Thus, its dynamics are very tightly linked to that of the butterfly and are not considered in this study. The generalist pupal parasitoid uses many Nymphalid butterfly hosts in Åland (Shaw et al. 2009) and is thus not restricted to the habitat patches suitable for the butterfly, and is also not considered in this study. The parasitoid wasp *C. melitaearum*, on the other hand, is limited to *M. cinxia* in Åland (Kankare et al. 2005) and exhibits patchy population dynamics. It has been surveyed systematically since 1997 (Ojanen et al. 2013), and is relatively rare in the landscape, most years inhabiting <10% of the host populations (Kahilainen et al. 2018). The wasp may increase the rate of local host extinction under rare circumstances (Lei and Hanski 1997), but generally does not (Kahilainen et al. 2018). Its population dynamics appear mostly constrained by the dynamics of the host (van Nouhuys and Hanski 2002, van Nouhuys and Lei 2004), its own sedentary behavior (Lei and Camara 1999), and strongly aggregating hyperparasitoids (van Nouhuys and Tay 2001).

The fungal pathogen *P. plantaginis* is a host‐specific obligate biotroph that completes its entire life cycle on the surface of the host plant, where it is visible as localized (nonsystemic) white powdery lesions. The pathogen is a significant stress factor for its host and may cause host mortality (Laine 2004*b*, Susi et al. 2015). The epidemiological dynamics in these populations have been studied since 2001 (Ovaskainen and Laine 2006, Ojanen et al. 2013), demonstrating that the fungal pathogen persists as a highly dynamic metapopulation through extinctions and (re‐)colonizations of local host populations (Jousimo et al. 2014). The first visible signs of infection appear in late June, and infection is transmitted both within and among host populations through July and August. There is potential for the mildew and butterfly to interact directly because lesions occur on the *P. lanceolata* leaves at the same time as butterfly larvae are feeding.

### Annual survey

Each autumn (August–September) the ~4,500 habitat patches suitable for the butterfly, as defined by the presence of one or both of its host plants, are systematically censused for occupancy and population size (number of winter nests) over an area of 50 × 70 km (Fig. 1). Field methods are exhaustively described in Ojanen et al. (2013). During each autumn census, all patches are searched for butterfly nests, and the presence and abundance of the mildew is recorded. Those patches occupied by the butterfly are revisited in the spring to assess the overwinter survival of the larval families. The presence of the parasitoid is also recorded at this time, when newly pupated wasps from the overwintering generation can be found in and around the silken host nests.

The abundance of the host plants is recorded during the autumn survey by visually estimating the area covered by each host plant species, and by assigning a categorical score between 0 and 3 where 0 indicates absence of the plant species, and 3 indicates substantial occurrence. For *P. lanceolata*, we used the visually estimated cover (in meters squared) as a measure of abundance. Plant cover data were not collected in 2009 and 2010, and we therefore used patch‐mean values for these years (see Jousimo et al. 2014). For *V. spicata*, cover was recorded in too few years to be included in the analyses, and we therefore used the categorical scale 0–3 as a measure of abundance.

### Weather conditions

Previous work suggests important effects of summer precipitation on the dynamics of our focal species (Hanski and Meyke 2005, Jousimo et al. 2014, Tack et al. 2015, Kahilainen et al. 2018). To incorporate these effects into our analyses, we extracted yearly precipitation data for each of the months May–August from the Finnish Meteorological Institute (Aalto et al. 2016).

### Species‐specific connectivity measures

We computed the species‐specific connectivity (*S*) of each patch *i* in year *t* for species *Y* as

$$S_{\mathrm{it}}^{Y}=\sum_{j\neq i}e^{‐\alpha d_{\mathrm{ij}}}\sqrt{A_{j}}O_{jt‐1}^{Y}$$

where *d_ij_* is the distance (in km) between patch *i* and patch *j*, $A_{j}$ is the area (in meters squared) of patch *j*, and $O_{jt‐1}^{Y}$ is the occupancy status of the focal species *Y* in patch *j* in year *t* − 1 (1 = occupied, 0 = not occupied). The term $e^{‐\alpha d_{\mathrm{ij}}}$ corresponds to the negative exponential dispersal kernel with scale parameter α. The inverse of the scale parameter (1/α) represents the average migration distance, which we assumed to be 1 km for all species. This value of the scale parameter corresponds well to estimates from mark–recapture studies and previous model estimates (see Hanski et al. 2017 for the butterfly, van Nouhuys and Hanski 1999 for the parasitoid, and Jousimo et al. 2014 for the mildew). We refer to the species‐specific connectivity measures as $S_{\mathrm{it}}^{M}$ for the butterfly, $S_{\mathrm{it}}^{C}$ for the parasitoid, and $S_{\mathrm{it}}^{P}$ for the mildew.

Previous work suggests that the resistance of *P. lanceolata* to mildew infection depends on the connectivity of the host populations (Jousimo et al. 2014). We therefore included a measure of host connectivity as

$$S_{i}^{\mathrm{PL}}=\sum_{j\neq i}e^{‐\alpha d_{\mathrm{ij}}}\sqrt{A_{j}^{\mathrm{PL}}}$$

where $A_{j}^{\mathrm{PL}}$ is the average (across years) coverage of *P. lanceolata* in patch *j*.

### Colonizations, extinctions, and patch states

We assigned colonization events when a species was present in a patch in year *t*, but absent in year *t* − 1, and extinction events when a species was absent from a patch in year *t*, but present in year *t* − 1. These definitions assume that a species present in a patch is detected, which is not always the case (Ojanen et al. 2013). Because surveys are not repeated within years, we cannot explicitly incorporate detectability in our analyses. However, as in previous analyses of these data (Hanski et al. 2017), we assumed that the influence on nondetection on the overall patterns was limited. Using these definitions, we assigned 3,976 (7.6%) colonization events and 4,546 (44.0%) extinction events for the butterfly, 2,950 (5.2%) colonization events and 2,718 (44.0%) extinction events for the mildew, and 301 (3.3%) colonization events and 317 (71.0%) extinction events for the parasitoid (percentages correspond to the number of events divided by the number of possible events, i.e., the sample size in the respective models). For the parasitoid, possible colonization events include colonizations of existing host populations, and joint colonizations by the host and its parasitoid. During the study period, 52 colonizations were joint with the host (i.e., both species colonized in the same year) and the rest were colonization of existing host populations. Similarly, 85 parasitoid extinctions were due to extinction of its host, and the rest were extinctions from persisting host populations.

We defined patch state as a categorical variable indicating the presence/absence of the butterfly *M. cinxia* (M), the parasitoid *C. melitaearum* (C), and the mildew *P. plantaginis* (P), respectively, where 0 means no species present, and MCP means all species present. Because the parasitoid cannot be present without its butterfly host, this yields six possible patch states (0, M, MC, P, MP, and MCP).

#### Analyses

We built our analyses around the assumption that, if our three focal species affect each other’s metapopulation dynamics, models assuming no such species interactions should fail to replicate the observed dynamics of the entire metacommunity. Although failure to reject the null hypothesis of species independence would not directly provide evidence against interspecific effects, this null‐model approach is conceptually useful by providing a benchmark against which to compare observed patterns (Peres‐Neto et al. 2001). We tested for deviations from species independence in two ways.

First, we included patch state in the previous year as a predictor for colonization and extinction rates of each species. An effect of patch state on the colonization or extinction rate of any of the focal species would mean that patterns of co‐occurrence affect the metapopulation dynamics of that species. Furthermore, because the transition probability between any two patch states can be written in terms of the species‐specific colonization and extinction rates (Fidino et al. 2019), an effect of patch state on any of the six rates also implies an effect on metacommunity dynamics. Focusing on the individual rates allows us to pinpoint which specific components of metacommunity dynamics are affected. Our focus on patch states in the previous year is motivated by the aim of testing the predictability of metacommunity dynamics (i.e., whether co‐occurrence patterns in the following year can be forecasted knowing the current occurrence patterns).

Second, we compared observed patterns of patch‐state distributions over time and space to simulations based on models assuming independence among the focal species’ metapopulation dynamics. This approach focuses on the overall patterns at the level of the metacommunity instead of patterns within individual patches, and is thus a complementary and more conservative test of deviations from the null‐model expectations.

### Modeling species‐specific colonization and extinction rates

We modeled species‐specific colonization and extinction rates by fitting generalized linear mixed‐effects models with binomial errors and logit link functions. Patch and year were treated as random factors. Thus, we modeled the colonization probability for species *Y* in patch *i* in year *t*, conditional on absence in year *t* − 1, as

$$c_{\mathrm{it}}^{Y}=1/\left(1+e^{‐\left(\beta_{0}^{Y}+\sum_{j}\beta_{j}^{Y}x_{\mathrm{ijt}}^{Y}+\gamma_{1t}^{Y}+\gamma_{2i}^{Y}\right)}\right)$$

and the extinction probability conditional on presence in year *t* − 1 as

$$e_{\mathrm{it}}^{Y}=1/\left(1+e^{‐\left(\beta_{0}^{Y}+\sum_{j}\beta_{j}^{Y}x_{\mathrm{ijt}}^{Y}+\gamma_{1t}^{Y}+\gamma_{2i}^{Y}\right)}\right)$$

where β_0_ is an intercept, β_*j*_ is the regression slope for covariate *j*, *x_ijt_* is the value of covariate *j* in patch *i* in year *t*, γ_1_ is a year‐specific random effect, and γ_2_ is a patch‐specific random effect. For the parasitoid, colonization is conditional on the presence of its butterfly host.

For the butterfly and parasitoid colonization and extinction models, fixed effects (i.e., the covariates *x^Y^* above) included the abundances of the plants *P. lanceolata* and *V. spicata*, the presence of roads bordering the patch, and the species‐specific connectivity (*S^M^* and *S^C^*, respectively). The mildew models were similar, but included the additional measure of host‐population connectivity (*S^PL^*), and did not include *V. spicata* abundance. Although these models include the potential biotic interactions involving plants, they do not consider potential butterfly–parasitoid–pathogen interactions. We included road presence in all of the models because previous studies have demonstrated an apparent role of roads as dispersal corridors for the mildew (Laine and Hanski 2006, Jousimo et al. 2014) and the butterfly (Schulz et al. 2019). For the weather variables, we performed model selection by comparing models fitted with precipitation data for all combinations of the months May–August (*n* = 2^4^ = 16 candidate models for the four weather variables), and selected the highest ranked models based on AIC values (Burnham and Anderson 2002). We obtained marginal and conditional *r* ^2^ values by the method of Nakagawa and Schielzeth (2013), and computed the variance explained by individual fixed effects as $\beta_{j}^{2}\sigma^{2}\left(x_{j}\right)$.

### Model‐predicted metapopulation and metacommunity dynamics

To evaluate the metapopulation dynamics of each species independently, we used the parameter estimates from the colonization and extinction models to predict the metapopulation dynamics of each species. A species is present in a patch in year *t* if it was present in year *t* − 1 and did not go extinct, or if it colonized in year *t*. For each yearly transition (from year *t* − 1 to year *t*), we computed the predicted occurrence probability (*p*) of each species *Y* in each patch *i* as

$$p_{\mathrm{it}}^{Y}=o_{t‐1}^{Y}\left({1‐e}_{\mathrm{it}}^{Y}\right)+\left(1‐o_{t‐1}^{Y}\right)c_{\mathrm{it}}^{Y}$$

where $o_{t‐1}^{Y}$ is the observed occupancy of species *Y* in year *t* − 1. To incorporate parameter uncertainty, we obtained 95% prediction intervals from 1,000 parametric bootstrap estimates drawn from the multivariate sampling distributions of the colonization and extinction models. We further incorporated uncertainty in the binary observation process by assigning presences and absences as random Bernoulli draws with the probability set to the predicted occurrence probability (*p_it_*).

To obtain community‐level predictions (“patch states”), we combined the species‐specific predictions for each patch–year combination with the following constraints. If the butterfly was predicted to go extinct, the parasitoid also went extinct. If the butterfly was predicted to colonize a given patch, we assigned parasitoid presence by sampling from the binomial distribution with the probability set to the estimated colonization probability for the focal patch. We then obtained predictions for temporal dynamics by summarizing these predictions for each year, and for a gradient of *P. lanceolata* cover by splitting patch cover into 15 equal‐size classes and summarizing the predictions for these (averaged across year).

### Assessing effects of interacting species on colonization and extinction rates

To assess whether colonization and extinction dynamics differed depending on the presence of interacting species, we fitted models identical to those described above but included patch state in year *t* − 1 as a fixed effect. To assess statistical support for different colonization and extinction rates depending on the presence of one or more potentially interacting species, and thus support for an effect of species interactions on metacommunity dynamics, we compared the models including and excluding patch state using AIC (Appendix S1: Table S1). To facilitate interpretation, we computed the predicted colonization and extinction rates for each of the possible patch states by inverse‐logit transforming the respective parameter estimates.

### Simulating metapopulation and metacommunity dynamics

To assess possible long‐term effects of species interactions further, we performed model‐based simulations of metapopulation and metacommunity dynamics based on the parameters of the “no interactions” models, and compared these predictions to the observed dynamics. We simulated 100 time series for the complete system, initiated from the observed patch occupancy patterns in year 2000. These simulations were performed as the predictions above, except that the predictions for each yearly transition were made based on the predicted patch occupancy patterns in each year; that is,

$$p_{\mathrm{it}}^{Y}=p_{t‐1}^{Y}\left({1‐e}_{\mathrm{it}}^{Y}\right)+\left(1‐p_{t‐1}^{Y}\right)c_{\mathrm{it}}^{Y}$$

where $p_{t‐1}^{Y}$ is the predicted occupancy of species *Y* in year *t* − 1. For each year, we recomputed the species‐specific connectivity measures ($S_{\mathrm{it}}^{M},S_{\mathrm{it}}^{C},S_{\mathrm{it}}^{P}$) based on the simulated occupancy patterns in the previous year. As above, we obtained community‐level predictions by combining the species‐specific predictions.

## Results

### Temporal metapopulation dynamics

**Fig. 2:**
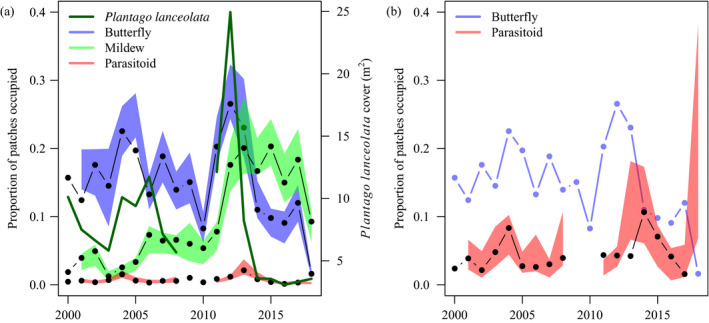
(a) Observed (black points and lines) and model‐predicted (colored 95% prediction intervals) metapopulation dynamics of the butterfly *Melitaea cinxia*, its parasitoid *Cotesia melitaearum*, and the powdery mildew *Podosphaera plantaginis*. The solid green line shows the mean patch cover by *Plantago lanceolata*, a host plant for both the mildew and the butterfly. Predictions are made for each yearly transition using the parameters from the species‐specific colonization and extinction models assuming no butterfly–parasitoid–mildew interactions. (b) Observed and model‐predicted metapopulation dynamics with the proportion of patches occupied by the parasitoid shown as a proportion of the predicted number of patches occupied by its butterfly host. [Color figure can be viewed at wileyonlinelibrary.com]

Since the start of the study period in year 2000, patch occupancy has fluctuated and weakly declined for the butterfly, increased for the mildew, and remained consistently low for the parasitoid (Fig. 2). Patch occupancy increased markedly for all species in 2012, in association with a peak in mean patch coverage of *P. lanceolata* (Fig. 2). Since 2013, the mean patch coverage of *P. lanceolata* has declined to less than half of the long‐term mean (8.03 m^2^). In 2018, patch occupancy by the butterfly, parasitoid, and mildew all declined dramatically, in association with a comparatively dry spring (May precipitation = 23.3 mm, 50.2% of the study‐period mean) and summer (July precipitation = 23.8 mm, 53.5% of the study‐period mean).

### Drivers of colonization and extinction probabilities

All species were more likely to colonize and less likely to go extinct from patches with greater coverage of *P. lanceolata* (Table 2). The butterfly and the parasitoid were also more likely to colonize and less likely to go extinct from patches with greater abundance of *V. spicata* (Table 2). The effect of *P. lanceolata* cover was stronger for the butterfly than for the parasitoid, and vice versa for the effects of *V. spicata* abundance. Specifically, *P. lanceolata* cover accounted for 13.8% of the explained variation in butterfly colonization and 15.3% of the explained variation in mildew colonization, but only 2.3% of the explained variation in parasitoid colonization. In contrast, *V. spicata* abundance accounted for 0.2% of the explained variation in butterfly colonization and 22.9% of the explained variation in parasitoid colonization.

**Table 2:** Parameter estimates ±SE for the highest‐ranked single‐species colonization and extinction models.

Response variable	Intercept (log odds)	*P. lanceolata* (log odds log m^−2^)	*V. spicata* (log odds *VS* ^−1^)	Connectivity (log odds *S* ^−1^)	Host connectivity (log odds *S* ^−1^)	Road presence (log odds)	Precipitation (log odds log mm^−1^)				*r* ^2^_M_	*r* ^2^_C_
							May	June	July	August		
*Melitaea cinxia*												
Colonization	−9.85 ± 0.49	0.69 ± 0.02	0.50 ± 0.03	0.96 ± 0.02		0.35 ± 0.06	0.22 ± 0.12				0.48	0.65
Extinction	1.82 ± 1.21	−0.66 ± 0.03	−0.34 ± 0.03	−0.59 ± 0.04		−0.32 ± 0.07			0.47 ± 0.22	0.42 ± 0.22	0.19	0.51
*Cotesia melitaearum*												
Colonization	−6.72 ± 0.91	0.20 ± 0.05	0.66 ± 0.06	0.17 ± 0.01		0.22 ± 0.16		0.21 ± 0.25			0.28	0.41
Extinction	1.58 ± 1.56	−0.35 ± 0.11	−0.49 ± 0.16	−0.06 ± 0.02		0.26 ± 0.33		0.52 ± 0.44			0.14	0.46
*Podosphaera plantaginis*												
Colonization	0.69 ± 0.85	0.75 ± 0.03	−	0.13 ± 0.02	−0.75 ± 0.06	0.72 ± 0.07		−0.47 ± 0.13		−0.42 ± 0.18	0.16	0.53
Extinction	1.60 ± 0.77	−0.39 ± 0.04	−	−0.14 ± 0.04	0.38 ± 0.04	−0.13 ± 0.09			−0.44 ± 0.20		0.05	0.37

Notes

*VS* is the abundance of *Veronica spicata* measured on an ordinal scale ranging from 0 to 3. *S* is a species‐specific connectivity measure (see Methods). *r* ^2^_M_ is the marginal *r* ^2^, which gives the proportion of variance explained by the fixed effects, and *r* ^2^_C_ is the conditional *r* ^2^, which gives the proportion of variance explained by the fixed and random effects combined. Random effects in all models are patch and year.

All species were more likely to colonize and less likely to go extinct from better‐connected patches, that is, those patches that were close to other occupied patches (Table 2). For the mildew, connectivity to all *P. lanceolata* patches (host connectivity; *S^PL^*) had stronger effects than the connectivity to those patches currently occupied by the mildew (*S^P^*). Both the butterfly and the mildew were more likely to colonize and less likely to go extinct from patches bordering roads. Precipitation patterns affected all colonization and extinction rates, although the effects were specific to each rate and sometimes poorly supported statistically (Table 2).

### Model‐predicted metapopulation and metacommunity dynamics

**Fig. 3:**
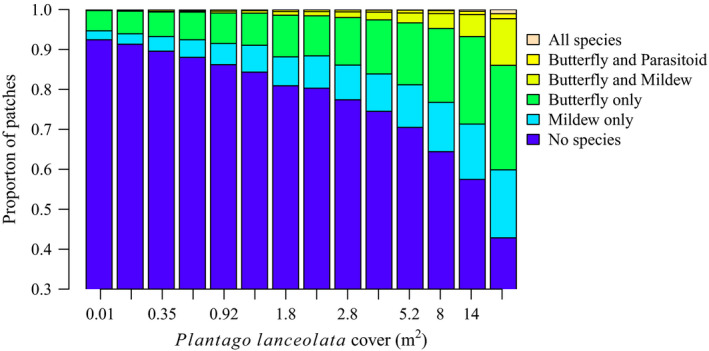
Predicted patch occupancy patterns based on the species‐specific colonization and extinction models assuming no butterfly–parasitoid–mildew interactions, along a gradient spanning 15 equal‐size classes of *Plantago lanceolata* cover. [Color figure can be viewed at wileyonlinelibrary.com]

The species‐specific models replicated well the observed metapopulation dynamics of each species (Fig. 2). The expected distribution of patch states varied markedly along the gradient of *P. lanceolata* cover (Fig. 3), with all species tending to occupy greater proportions of those patches where *P. lanceolata* was more abundant.

### Effects of interacting species on colonization and extinction rates

**Fig. 4:**
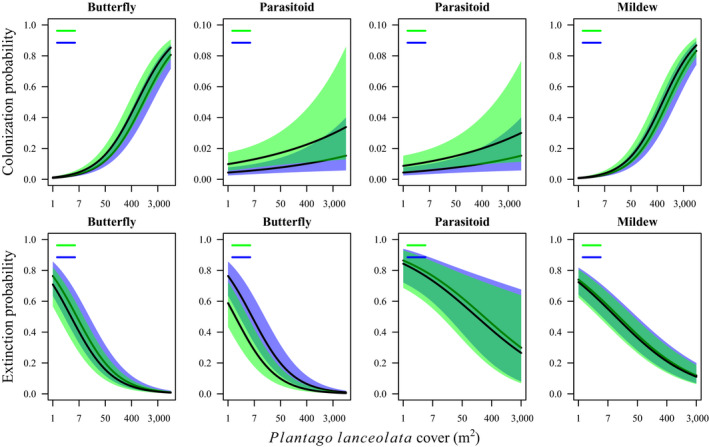
Response curves with 95% confidence intervals illustrating the estimated effect of host plant abundance on the colonization and extinction probabilities of the butterfly *Melitaea cinxia*, the parasitoid *Cotesia melitaearum*, and the powdery mildew *Podosphaera plantaginis*, in patches where potential interacting species are present vs. absent. For the parasitoid, colonization of patches currently unoccupied by its host implies joint colonization of a patch by both species. [Color figure can be viewed at wileyonlinelibrary.com]

The probabilities of colonization and to a lesser extent extinction differed depending on which species were present in the focal patch the previous year (Fig. 4, Table 3). For all three species, colonization was least likely into patches currently unoccupied by any species, and the probability of colonization increased with the number of potential interacting species present. For example, the butterfly was more likely to colonize and less likely to go extinct from patches occupied by the mildew, and even less likely to go extinct from patches occupied also by its parasitoid. The mildew was somewhat more likely to colonize patches occupied by the butterfly, and even more likely to colonize patches occupied by both the butterfly and its parasitoid. The parasitoid was most likely to colonize patches currently occupied by both the butterfly and the mildew. Finally, we detected no effect of patch state (occupancy by the other species) on the extinction probabilities of the mildew or the parasitoid (Table 3).

**Table 3:** Effects of patch state in the previous year on species‐specific colonization and extinction rates given in % (i.e., ×100) with 95% confidence intervals.

Response variable	Patch state in previous year						ΔAIC
	0	M	MC	MCP	MP	P	
*Melitaea cinxia*							
Colonization	1.96 (1.34, 2.85)					2.70 (1.82, 4.00)	−15.7
Extinction		50.6 (36.8, 64.3)	31.9 (19.6, 47.4)	29.1 (15.2, 48.3)	43.4 (30.1, 57.8)		−32.5
*Cotesia melitaearum*							
Colonization	0.58 (0.34, 1.00)	1.30 (0.81, 2.08)			1.79 (1.01, 3.15)	1.15 (0.48, 2.74)	−20.1
Extinction			75.7 (58.4, 87.3)	72.4 (51.3, 86.8)			1.8
*Podosphaera plantaginis*							
Colonization	1.73 (1.15, 2.60)	2.36 (1.55, 3.58)	3.71 (2.08, 6.54)				−26.6
Extinction				61.1 (45.0, 75.1)	57.7 (46.5, 68.2)	59.7 (48.9, 69.7)	2.8

Notes

Estimates were obtained while holding all environmental covariates (described in Table 2) constant at their means. The patch state indicates the presence/absence of *M. cinxia* (M), *C. melitaearum* (C), and *P. plantaginis* (P), respectively, with 0 indicating no species present, and MCP indicating all species present. The column ΔAIC gives the difference in AIC between a model including patch state as a fixed effect, and a similar model excluding patch state, with negative values indicating support for the full model including patch state (see Table S1 for the complete model comparison).

### Simulated metacommunity dynamics

**Fig. 5:**
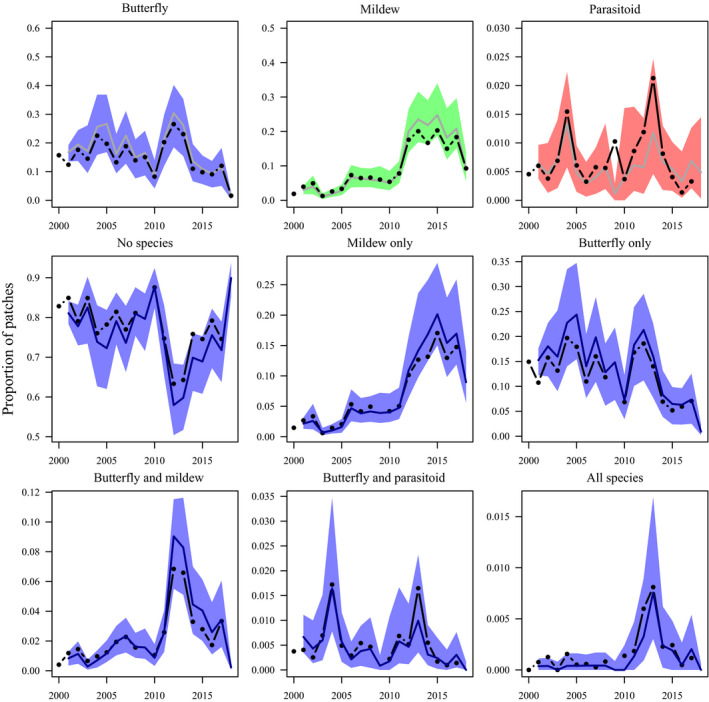
Observed (black dots and lines) and simulated (with 95% prediction intervals) metapopulation (top row) and metacommunity dynamics of the butterfly *Melitaea cinxia*, its parasitoid *Cotesia melitaearum*, and the powdery mildew *Podosphaera plantaginis*. Simulations were run based on the parameters of the species‐specific colonization and extinction models assuming no butterfly–parasitoid–mildew interactions, and initiated from the observed patch states in 2000. [Color figure can be viewed at wileyonlinelibrary.com]

The simulations based on the “no interactions” models replicated reasonably well both the observed single‐species metapopulation dynamics, and the observed metacommunity dynamics (Fig. 5). Consequently, there was no strong evidence that the dynamics of the metacommunity differ from what could be expected based on the independent metapopulation dynamics of each species.

## Discussion

The dynamics of ecological communities are expected to depend in part on species interactions within and among trophic levels, which can be studied by analyzing joint time series of potentially interacting species that share a common habitat network. In the Åland islands, the structure of the dry‐meadow metacommunity module comprising the butterfly *M. cinxia*, its host plants *P. lanceolata* and *V. spicata*, its parasitoid wasp *C. melitaearum*, and the fungal plant pathogen *P. plantaginis* infecting *P. lanceolata* has changed markedly over the last 20 yr. Following a peak year for the host plant *P. lanceolata*, the butterfly, and the mildew in 2012, the size of the butterfly metapopulation has declined while the size of the mildew metapopulation has remained high, and the mildew is currently more abundant across the landscape than is the butterfly. However, despite abundant previous work showing that these species affect each other’s individual performance (Table 1), we detected limited evidence that interactions among the insects and pathogen influence their metacommunity dynamics over time and across the landscape. Although the analyses suggested that colonization and to a lesser extent extinction rates are related to the presence of interacting species within patches, the directionality of these patterns were mostly contrary to expectations (Table 1). Furthermore, simulations assuming species independence replicated reasonably well the observed metacommunity dynamics, suggesting that the influence of species interactions on metacommunity dynamics are weak compared to other environmental drivers and thus difficult to detect in observation data. In contrast, all species responded to variation in plant abundance, suggesting important effects of variation in host plant abundance on the entire metacommunity module.

### Drivers of metapopulation dynamics across a heterogeneous landscape

As a key step in testing for a signal of species interactions in the dynamics of the insect–pathogen metacommunity, we first modeled the dynamics of each species separately, thus building an environmental “null model” of the system (Peres‐Neto et al. 2001). The observed positive effects of host plant abundance and connectivity on colonization and negative effects on extinction probabilities of each species are consistent with predictions of metapopulation theory (Hanski and Gaggiotti 2004), with previous analyses of different subsets of the time‐series data for each species in this system (van Nouhuys and Hanski 2002, Jousimo et al. 2014, Hanski et al. 2017), and with findings in other systems (Weisser 2000, Antonovics 2004, Johansson et al. 2012). The increased colonization and reduced extinction rates in patches bordering roads (see also Jousimo et al. 2014, Schulz et al. 2019, Numminen and Laine 2020) may relate to the role of roads as dispersal corridors, mediated by the presence of scattered host plants along roads. Although roads are known to cause mortality of dispersing butterflies (Munguira and Thomas 1992), car traffic is very low in most of Åland, and open road verges are known to increase dispersal for some butterfly species (Öckinger and Smith 2007, Skorka et al. 2018). This suggests that similar responses of several disparate species, with contrasting dispersal traits, to landscape features such as permeability can contribute to species co‐occurrence and thus affect metacommunity dynamics (see e.g., Jones et al. 2015, Guzman et al. 2019).

The observed effects of early‐ vs. late‐summer precipitation on colonization and extinction dynamics were idiosyncratic across species, but broadly consistent with previous work suggesting important effects of summer precipitation on the dynamics of our focal species (Hanski and Meyke 2005, Jousimo et al. 2014, Tack et al. 2015, Kahilainen et al. 2018). A recent analysis of the 2018 population crash of the butterfly suggested that vegetation drying associated with dry weather led to greater extinction rates (van Bergen et al. 2020). In the current analysis we controlled for among‐year differences and detected an unexpected positive effect of July precipitation on butterfly extinction probability, suggesting that regional‐scale summer precipitation patterns may be a rather poor predictor of autumn plant abundance across the landscape.

### Limited influence of species interactions on metacommunity dynamics

The colonization dynamics of all species, and the extinction dynamics of the butterfly, differed detectably depending on which other species were currently present in the focal patch. Because these effects were detected while controlling for environmental and spatial factors including the host plants, the naïve interpretation is that species interactions determine the probabilities of colonizations and, for the butterfly, extinctions. However, these effects could also represent joint responses of the species to unmeasured aspects of the environment (Ovaskainen et al. 2017*b*, Fidino et al. 2019). As an additional test of species nonindependence, we simulated metacommunity dynamics based on the parameters of the models assuming no interactions among the focal species. Failure of “neutral” models assuming species independence to replicate observed metacommunity dynamics would provide support for species interactions as important drivers of metacommunity dynamics. The fairly unbiased predictions of patch states obtained from combining species‐specific predictions (Fig. 5) are therefore consistent with independent dynamics of each species. These results do not provide direct evidence for species independence, though, because the observed patterns could match neutral expectations due to, for example, multiple interactions (among the focal species or involving other species not considered here) canceling out at the level of the entire metacommunity. Furthermore, the strong effects of environmental drivers and host‐plant abundance may have reduced the probability of detecting comparatively weak effects of host–parasitoid and insect–pathogen interactions.

The interspecific associations inferred from the patch‐state effects were always positive, and it is hard to imagine how, for example, the presence of the parasitoid would reduce the probability of its butterfly host going extinct (see Lei and Hanski 1997). A more parsimonious explanation for this association is that the parasitoid tends to occupy butterfly populations that are comparatively large and stable, and thus rarely go extinct (van Nouhuys and Hanski 2002). The butterfly and mildew interact through their shared host plant *P. lanceolata*. Previous work has found a negative effect of mildew infection on the growth and overwintering survival of butterfly larvae (Laine 2004*a*, Rosa et al. 2018), as well as on the growth rate of *P. lanceolata* populations (Penczykowski et al. 2015). Thus, the observed reciprocal positive effects of these two species on each other’s probability of colonization are again hard to explain as the outcome of positive interspecific interactions. If competitive interactions are important, we would expect negative effects of competitor occurrence on colonization and/or persistence, as observed by Dubart et al. (2019) for competing snail species. Both the mildew and butterfly larvae tend to cluster spatially within the host plant populations (Ovaskainen and Laine 2006, Salgado et al. 2020) and field observations suggest that they tend to occupy different parts of shared patches, which perhaps reduces any negative interaction between them. Moreover, it has been shown experimentally that butterfly larvae actively leave mildew‐infected host plants (Laine 2004*a*). Regarding the parasitoid and mildew, van Nouhuys and Laine (2008) suggested that a positive association could arise because mildew infection of *P. lanceolata* appears to lead to female‐biased sex ratios of the parasitoid, and thus an increased potential population growth rate. Overall, however, these observations lead us to propose that the observed effects of patch state on colonization and extinction dynamics represent, to a large extent, shared positive responses of the species to some unmeasured aspect of the environment, that outweighed any local negative interactions among them. We suspect that some of this variation relates to local‐scale variation in weather affecting the host plants, and possibly to heterogeneity within patches leading to spatial partitioning of resources between the butterfly and mildew.

Our focus on colonization–extinction dynamics was motivated by testing the predictive power of simple mechanistic models, which have been successful in classic metapopulation theory (Hanski and Gaggiotti 2004). However, ignoring variation in abundances of species almost certainly reduces the probability of detecting relatively weak interactions among them (Blanchet et al. 2020). Similarly, our focus on forecasting colonizations and extinctions from patterns of patch occupancy in the previous year was motivated by assessing the predictability of observed dynamics, but does not directly test for interactions within years, which may be important, for example, for herbivorous insects sharing their host plant with pathogens (Biere et al. 2013).

### Do host‐plant dynamics drive variation in the metacommunity?

In contrast to the weak influence of the dynamics of the butterfly, parasitoid, and mildew on one another, all species depended in important ways on spatial and temporal variation in the abundance of the host plants (or the butterfly’s host plants for the parasitoid). Interestingly, although the dynamics of both the butterfly and its parasitoid depended on the abundances of both host‐plant species, the butterfly responded more strongly to variation in *P. lanceolata* abundance, and the parasitoid responded more strongly to variation in *V. spicata* abundance. This positive association could arise if both *V. spicata* and the parasitoid respond to a common environmental driver, or as a consequence of a multitrophic interaction. There is support for the latter mechanism. Parasitoids must locate butterfly larvae by finding their host plant, which they are known to do primarily by using volatile cues produced by herbivore‐infested plants (Vet and Dicke 1992). The volatile mix produced by *V. spicata* changes when *M. cinxia* feeds on it (Pinto‐Zevallos et al. 2013), and its odor is more attractive to parasitoids than that of herbivore‐infested *P. lanceolata* (Castelo et al. 2010), which may lead to the observed greater parasitism of butterfly nests on *V. spicata* than on *P. lanceolata* (van Nouhuys and Hanski 1999).

We detected a spatial pattern in the mildew population dynamics as reflected by the mildew‐specific connectivity measure ($S_{\mathrm{it}}^{P}$), yet this effect was weaker than the corresponding effect for the butterfly. Furthermore, we detected much greater spatial variation unexplained by the environmental covariates for the mildew than for the butterfly (Table 2, *r* ^2^_C_ vs. *r* ^2^_M_, where *r* ^2^_C_ includes the variation explained by the random effects for patch and year). These results could reflect greater dispersal limitation for the mildew compared to the butterfly, or the response of the mildew to unmeasured aspects of the environment. The former is consistent with the emerging importance of “spatial use properties” of species in structuring metacommunity dynamics (Leibold et al. 2004, Guzman et al. 2019). Moreover, the interaction between the mildew and *P. lanceolata* is characterized by a high degree of local specificity, with infection outcome determined by genotype‐by‐genotype interactions (Laine 2011). Indeed, the metapopulation dynamics of the mildew depended on the connectivity of the host plant populations, consistent with an effect of host‐plant resistance on pathogen metapopulation dynamics (Jousimo et al. 2014). Although we have so far considered the abundance of the host plants as a key driver of population and community dynamics, it is clear that the genetic diversity within and among plant populations can affect the population dynamics of associated organisms (Hughes et al. 2008, Underwood 2009, Moreira and Mooney 2013) and potentially community structure (Crutsinger et al. 2006).

The strong effect of host‐plant abundance on all species suggests that the observed decline in *P. lanceolata* abundance since 2013 (Fig. 2) could have profound effects on the entire metacommunity. The apparent decline in *P. lanceolata* abundance may be partly due to land‐use changes or natural succession leading to shrub encroachment of the focal patches. A recent analysis using satellite‐derived vegetation indices demonstrated reduced productivity associated with the severe summer drought of 2018 (van Bergen et al. 2020), providing a mechanistic link between climatic variation and host‐plant availability. Dramatic population fluctuations and declines are common in temperate butterflies, and may often relate to fluctuations in host plant availability (Curtis et al. 2015). For example, the decline of the related Marsh fritillary (*Euphydryas auridia*) in Denmark has been linked to reduced host‐plant availability (Brunbjerg et al. 2017), and the *M. cinxia* metapopulation on the Isle of Wight seems to decline when cold summers reduce the availability of *P. lanceolata* in suitable condition (Curtis et al. 2014). Compared to the extensive literature on butterfly populations, less is known about the effect of climatic patterns on the metapopulation dynamics of parasitoids and fungal pathogens. The general view is, however, that species at higher trophic levels are more vulnerable than their hosts to changes in habitat quality (Cronin and Reeve 2005, Nair et al. 2016), some of which is driven by climate change.

## Conclusions

Our analyses of a metacommunity module surrounding the plants *P. lanceolata* and *V. spicata* in the Åland islands reveal only limited influence of species interactions on the spatiotemporal dynamics of interacting insect and pathogen metapopulations. In contrast, we detected consistent strong effects of plant abundance on insect and pathogen metapopulation dynamics, which in turn allowed us to obtain reasonably accurate predictions of metacommunity dynamics from models ignoring direct insect–insect and insect–pathogen interactions. Although experimental data make it clear that our three focal species are affecting each other at some level (Table 1), these effects do not appear to lead to detectable deviation from expected independent metapopulation dynamics. This suggests that other drivers of their population dynamics overshadow any effect of species interactions at the scale of metapopulation dynamics as described by extinction and colonization events. Our analyses would not, however, detect more nuanced effects on, for example, population size or local population growth rate (Kahilainen et al. 2018). Furthermore, an emerging insight from the long‐term study of the Åland metacommunity is that, although the impact of species interactions on metacommunity dynamics may be weak, interacting species can still influence one another by influencing patterns of genetic variation (Nair et al. 2016), and by imposing “soft” selection leading to evolution, as appears to occur, for example, in the interaction between the mildew and its host plant (Jousimo et al. 2014). These findings underline the importance of considering the effects of species interactions on evolutionary processes in long‐term, integrated studies of natural population dynamics.

## Supporting information

Appendix S1

## Data Availability

Data and associated R code are available on Zenodo. https://doi.org/10.5281/zenodo.3956435
