# Supplementary material for: Host‐plant availability drives the spatiotemporal dynamics of interacting metapopulations across a fragmented landscape
Source: Ecology. 2020 Oct 7;101(12):e03186. doi: 10.1002/ecy.3186 (PMC7757193; doi:10.1002/ecy.3186)
Supplement: Supplementary file 1 — Appendix S1 [file ECY-101-e03186-s001.pdf]

Supporting Information. Opedal, Ø.H., O. Ovaskainen, M. Saastamoinen, A.-L. Laine, and S. van Nouhuys. 2020. Host plant availability drives the spatio-temporal dynamics of interacting metapopulations across a fragmented landscape. Ecology.

Appendix S1

Table S1. Model comparison for colonization and extinction rates of the Glanville Fritillary butterfly *Melitaea cinxia*, its parasitoid wasp *Cotesia melitaearum*, and the fungal pathogen *Podosphaera plantaginis*. The 'patch-state' models include patch state (indicating which species are present) as a fixed covariate, while the 'no patch-state' models are the selected null models with no patch state effect. logLik is the log likelihood, k is the number of model parameters, AIC is the Akaike Information Criterion and ΔAIC is the difference in AIC between the two models.

Response variable	Model	logLik	k	AIC	ΔAIC
Melitaea cinxia					
Colonization	Patch state	-10430.2	9	20878.45	0
	No patch state	-10439.1	8	20894.19	-15.74
Extinction	Patch state	-5541.2	13	11108.44	0
	No patch state	-5560.5	10	11140.92	-32.48
Cotesia melitaearum					
Colonization	Patch state	-924.6	11	1871.27	0
	No patch state	-937.7	8	1891.38	-20.11
Extinction	Patch state	-213.8	9	445.57	0
	No patch state	-213.9	8	443.81	1.76
Podosphaera plantagini					
Colonization	Patch state	-9428.1	11	18878.14	0
	No patch state	-9443.4	9	18904.72	-26.58
Extinction	Patch state	-3573.9	10	7167.78	0
	No patch state	-3574.5	8	7164.94	2.84
